# Epigallocatechin-3-Gallate Toxicity in Children: A Potential and Current Toxicological Event in the Differential Diagnosis With Virus-Triggered Fulminant Hepatic Failure

**DOI:** 10.3389/fphar.2019.01563

**Published:** 2020-01-29

**Authors:** Consolato M. Sergi

**Affiliations:** ^1^ National “111” Center for Cellular Regulation and Molecular Pharmaceutics, Key Laboratory of Fermentation Engineering, Ministry of Education, Hubei University of Technology, Wuhan, China; ^2^ Stollery Children's Hospital, University Alberta Hospital, Edmonton, AB, Canada; ^3^ Department of Laboratory Medicine and Pathology, University of Alberta, Edmonton, AB, Canada; ^4^ Department of Orthopedics, Tianyou Hospital, Wuhan University of Science and Technology, Wuhan, China

**Keywords:** green tea, toxicity, hepatotoxicity, acute liver failure, pediatrics, metabolism, pharmacology, subcellular environment

## Abstract

The use of nutraceuticals is considerably increasing worldwide with a demand for organic and clean foods in the last two decades, which is probably incomparable with other periods of our civilization. The consistent application of nutraceuticals and so-called “superfood” may have remarkable effects on the prevention of several chronic diseases, including cancer. Moreover, the increased rate of overweight and obesity in Western countries does not spare childhood and youth, and the number of parents using natural remedies for preventing pediatric illness is vastly increasing worldwide. However, the overwhelming effects on diseases often overshadow the side effects of such nutrition, particularly in societies without millennial experience with botanicals and natural elements. Thus, the final result may be disastrous for some individuals. The liver is the most important and conspicuous target organ of numerous molecular compounds, and the cell damage is particularly striking on the infantile and pediatric liver due to the immaturity of the hepatocytes. Here, we target some generic data on fulminant hepatic failure, the benefits, and toxicity of epigallocatechin-3-gallate, which is one of the major components of green tea, and the histopathology of the “green-tea”-associated liver disease.

## Introduction

Nutraceuticals are compounds collectively defined under a broad umbrella term. These compounds are mostly derived from food sources and exhibit some health benefits other than the essential nutritional value. The benefits are variable and address the promotion of a general better well-being. Nutraceuticals also spawn discussions and concepts how to control illness and prevent malignancy. Some of these benefits are scientifically substantiated, while some are probably non-scientifically proved and lack solid scientific knowledge (Ward et al., 2019). Data using magnesium sulfate, sulforaphane (broccoli sprouts), creatine, choline, melatonin, and resveratrol in animal models suggest that natural health products may provide safe, effective, accessible, and affordable prevention of fetal brain injury and resulting lifelong disabilities (Shaw and Yager, 2019). Currently, some nutraceutical claims have also a trend. In some societies, they are also becoming part of philosophy with intransigent views that may mirror the appearance of a cult. One note that is often missed in healthcare reports on social media is the lack of a balance with the presentation of side effects with the beneficial effects (Abdualmjid and Sergi, 2013). This aspect is crucial mainly in vulnerable populations, such as children and youth (Pignatelli and Basili, 2010; Cicero and Colletti, 2018). Some societies, particularly in the West that have not been exposed to such compounds for hundreds of years, may leave individuals to adopt diet supplements in a state that may be more harmful than beneficial at some point. In the last century, this phenomenon was poorly known. Currently, several deaths of children whose parents had refused conventional therapy have stricken our society and, to some degree, the relationship between pediatricians and families (Krautkramer, 2005; Committee On B., 2013). In this paper, we target the virus-triggered fulminant hepatic failure and the hepatotoxicity of green tea and/or green tea extracts (e.g., epigallocatechin-3-gallate, EGCG) showing some histopathology data of “green-tea”-associated liver disease. Although EGCG is the major component in green tea, EGCG is not equal to green tea. Green tea is safely used in oriental countries without concerns about hepatotoxicity. It is important to emphasize that the green tea extract sold in the United States and western countries is not usually the “real” extract from green tea. There is, often, an addition of EGCG into the extract of green tea. The EGCG contents in such green tea extracts are remarkably higher than the original extract of green tea.

## Viruses-Trigged Fulminant Hepatic Failure

**Figure 1:**
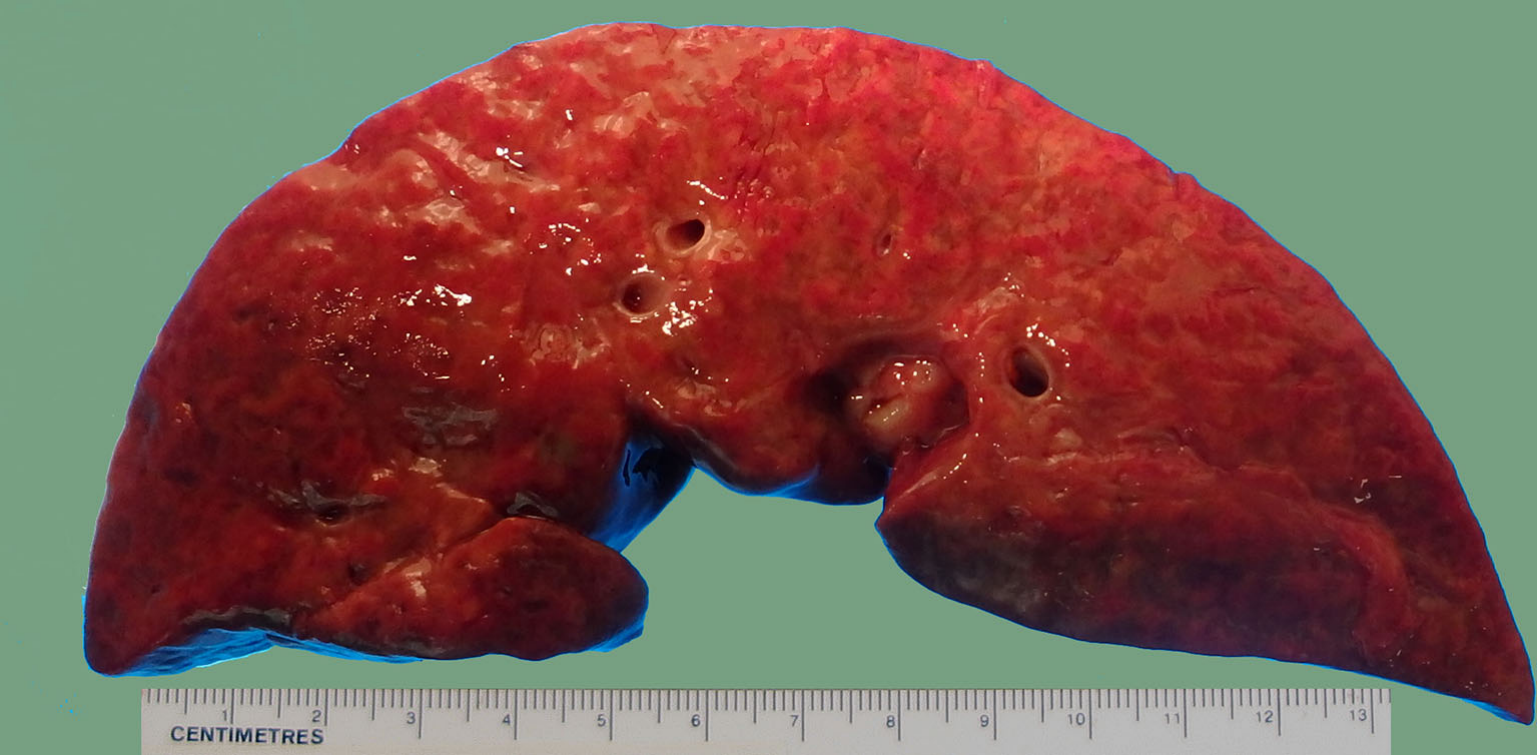
Gross photograph of a liver with fulminant hepatic failure. Note the bosselated appearance with wrinkling of the capsule and mottling cut surface due to intermingled red and yellow areas. No atresia of the extrahepatic biliary system was found by careful examination of the hilum.

Fulminant hepatitis with acute failure of the liver or academically known as fulminant hepatic failure (FHF) is a rare event in childhood, although it is not minimal (Fargion et al., 1996a; Sergi et al., 1999). FHF is a clinical syndrome. It is initially defined by the occurrence of encephalopathy of hepatic origin (hepatic encephalopathy) in a patient without pre-existing liver disease within a specific timeframe, i.e., eight weeks after onset of the first clinical symptoms (Mendizabal and Silva, 2016; Rosenblatt and Brown, 2018). Although FHF definition has changed over the years, FHF may still be distinguished on clinical grounds. Epidemiological and statistical studies have revealed that there are still prognostic implications in subdividing FHF in hyperacute, acute, and subacute liver failure, depending on whether the mentioned above interval is 7 days, 28 days, or 12 weeks, respectively (O'Grady et al., 1993). The gross morphology and histopathology of the “failing” (terminal) liver are standard and not necessarily point to a specific etiology. In all zones of the hepatic lobulus there is massive necrosis of hepatocytes, although periportal sparing of hepatocytes may be typical. Reticulin collapse and biliary duct proliferation are standard and accompanied by minimal or mild inflammatory reaction. There are a plethora of etiologic factors that can be considered responsible for triggering FHF. On several occasions, viruses, drugs, toxins, and metabolic disorders play a significant role (Abdualmjid and Sergi, 2013; Kodali and McGuire, 2015; Mendizabal and Silva, 2016; Garoufalia et al., 2019). In youth and adulthood, particularly in Asian countries, where the hepatitis B virus (HBV) vaccination has not been universally implemented, and the hepatitis A virus (HAV) is not considered to be implemented in healthcare, viruses play almost certainly a significant role in FHF. Since the identification of hepatitis C virus (HCV) as the leading agent for chronic post-transfusional and sporadic Non-A, Non-B (NANB) liver disease, antagonistic data as to its role in FHF have been divulgated in the scientific literature with the highest incidences reported in Japan and Taiwan (Sergi et al., 1998). During the dramatic course of FHF, there are numerous tests and false-negative results in testing only a single serum sample or “false positive” results due to virus uptake with untested blood products have been suggested to clarify these discrepancies (Sergi et al., 1996; Sergi et al., 2002). Also, it has been suggested that the examination of hepatic tissue from the explanted organ of subjects with NANB FHF undergoing liver transplantation may overthrow this health issue. Another critical aspect to consider is the combination of viral infections with the augmented trigger effect on the liver, which is entirely or submassively necrotic in the event of liver failure with a fulminant course (**Figure 1**).

Consequently, ruling out HBV induced FHF from investigations targeting the role of HCV in FHF may lead to miscalculating the role of this virus in FHF, but this aspect is also debated. Long-term prognosis and early post-liver transplantation may be jeopardized if an overlooking of a viral infection occurs. Recurrence of the original disease can be a relentless problem in the differential diagnostic procedures. As with HBV, the same setting may be valid for the hepatitis G virus (HGV). This virus has been found in chronic HCV infected patients (Sergi et al., 1998; Summerfield, 2000; Noor et al., 2018). Other uncommon viral etiologic factors that should be considered are cytomegalovirus (CMV) and parvovirus B19 (PVB19). In particular, CMV may both trigger an FHF and biliary atresia in infants (Spearman et al., 2005; Sergi, 2019). These etiologies may be obviously different according to the geographic area. Thus, FHF remains a challenging area in pediatric hepatology and is under investigation for formulating better guidelines worldwide.

## Nutraceutical Use

Nutraceuticals can be a cause of FHF at any age (Karvellas et al., 2016; Cicero and Colletti, 2018; Stravitz and Lee, 2019). The exclusion of viral etiologic factors should suggest a toxic, autoimmune, or metabolic disorder as the culprit for FHF (Rosenblatt and Brown, 2018). A thorough clinical history among the patients and their parents is mandatory. The use of nutraceuticals is considerably increasing worldwide with demand in organic and clean foods, which is now seen in pediatrics and probably considered incomparable with other periods. The consistent application of nutraceuticals and so-called “superfood” has remarkable and tangible experiences in the prevention of numerous chronic non-oncological diseases and tumors. Also, the increased rate of obesity in Western countries does not spare childhood and youth, and the number of parents using nutraceuticals and natural remedies for pediatric obesity or illness is vastly increasing worldwide (Brunt, 2004; Hartman and Shamir, 2012). Sadly, the side effects of such nutrition are often overshadowed on both professional publications and social media. The resurgence of nutritional rickets in the 21^st^ century may be associated, at least partially, to some vegan and vegetarian diet imposed on children by parents belonging to specific communities, no matter which faith they profess (Zhang et al., 2016; Antonucci et al., 2018; Yang et al., 2019). The histopathology of “green tea” or better green tea extracts-associated toxicity in children is poorly known. The potential dreadful event of FHF may need to be emphasized and extensively discussed in both professional and social media. Currently, pediatric obesity is a tragic evolution of the individual, and the use of social media and videogames have worsened the inactivity of youth worldwide. Obesity, which results from a positive energy balance, may represent an inexorable health problem, particularly in Western countries, but Asian countries, including China, have shown an increased rate in the last decade (Dong et al., 2019; Butler et al., 2019). Many children and youth wish to use one solution, and de-tox teas, as well as chemical compounds, are continually popping up in magazines and social media as well as in teenager events (Floyd, 2009; Cline, 2015). Obesity and wrong information on it, practically not curated by professional medical associations, is now found across well-developed and underdeveloped countries worldwide. Recently, the World Health Organization (WHO) appraised that more than 1.5 billion individuals with age more than 18 years worldwide are overweight. At least 500 million of which should be considered obese (Hartman and Shamir, 2012; West-Eberhard, 2019). The number of parents using nutraceuticals and natural remedies for pediatric illness and obesity is fast increasing, and some of these parents may also belong to the anti-vaxxer group, which is considered a new group of individuals against vaccination schedules (Signorelli, 2019). These groups may facilitate the recrudescence of exanthemata, which we considered almost disappeared (Leung et al., 2018; Leung et al., 2019). Although not comprehensive, we explored some studies that have been completed to characterize potential hazards following the consumption of green tea and/or green tea extracts. The liver is the target organ of this toxicity. The cell damage of EGCG, the major component of green tea, or similar components, to the infantile and pediatric liver may have fatal consequences.

## Green Tea

In consideration of the increasing use of nutraceuticals in childhood and several youth pathologies, we performed a critical review of toxicology to portray potential hazards following the ingesting of green tea and its extracts. Hepatotoxicity is life-threatening and has been associated with certain dosing conditions. Tea (*Camellia sinensis*, *Theaceae*) is a favorite beverage and consumed by over two-thirds of the world population, particularly in China, India, Japan, and countries of the Commonwealth (Noor et al., 2018). The tea market is dominated by black tea, which is the kind of tea most largely traded worldwide (about 80%). The black tea is prevalent in North America and Europe, while the green variety is primarily consumed in mainland China, Taiwan, and Japan. The rarest tea used is white tea, which is also the least handled. The Oolong tea (semi-fermented) is a rare tea, which is in the middle between the fermented (black tea) and non-fermented tea (green tea). Three additional types of teas are also known. They include scented tea, compressed tea, and organic tea. In the scented tea, the additional flavoring is mixed to the non-/semi-/or fermented leaves as a final stage, while compressed tea is bricks of processed tea leaves that have been hydraulically compressed. In the production of organic tea, whose production started twelve to thirty years ago, the cultivation of tea follows strict rules which are controlled by organic agencies.

## Green Tea Polyphenols (GTPs)

**Figure 2:**
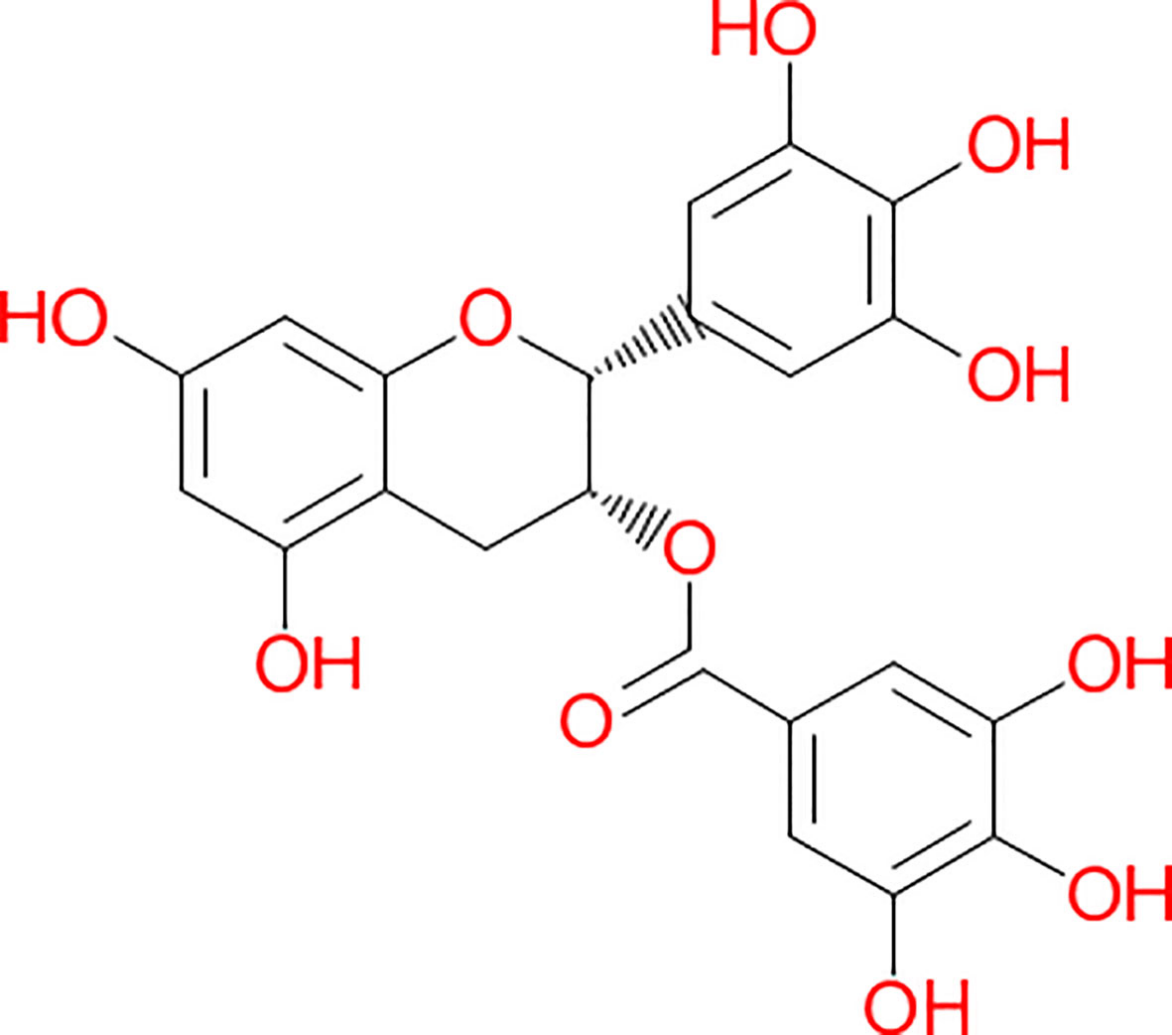
Chemical structure of (-)-Epigallocatechin-3-gallate. This structure was derived by the application of the software Chem4Word 3.0, United Kingdom.

The major bioactive molecules of tea (30–42% of tea leaf dry weight) are the polyphenols in addition to methylxanthine alkaloids (caffeine, theophylline, and theobromine) (Balentine et al., 1997; Mazzanti et al., 2015). The group of the polyphenols contains the catechin, EGCG (**Figure 2**), along with epicatechin (EC), epigallocatechin (EGC), and epicatechin-3-gallate (ECG). EGCG is the most copious component reaching up to 80% of total content of polyphenols (Balentine et al., 1997; Ishii et al., 2011). Chemically, it is the ester of epigallocatechin and gallic acid. Polyphenols, which are also known as polyhydroxyphenols, are a structural class of mainly natural, but also synthetic or semisynthetic, organic molecules. Polyphenols contain large multiples of phenol structural units. EGCG is being used in many dietary supplements. According to the United States Department of Agriculture (USDA) Database including the Flavonoid Content of Selected Foods, EGCG is found in high content (7.4 g per 100 g) in the dried leaves of green tea, while almost half of this compound is present in white tea (4.2 g per 100 g). A decrease of 87.3% of this compound is found in black tea (0.9 g per 100 g) (Bhagwat et al., 2011). EGCG possesses the highest antioxidant potential and, to the best of our knowledge, is by far the most biologically active substance. The antioxidant properties with their protective roles against pathologies associated with a considerable amount of reactive oxygen species (ROS), such as cardiovascular diseases and cancer, are ascribed to the high catechin levels contained in the tea, particularly the green variety (Rietveld and Wiseman, 2003). The fermentation of the tea leaves induces the oxidization of catechins by polyphenol oxidase. The catechins are, then, changed into theaflavins and thearubigins. These chemical compounds are condensed polymeric molecules, which have been considered accountable for the typical organoleptic features of black tea. Non-fermented tea leaves only constitute the green tea, which is the primary source of catechins. EGCG has demonstrated poor oral absorption rates even considering a daily intake corresponding to 8–16 cups of green tea. Moreover, bioavailability investigations indicate that EGCG blood levels peak within 1.7 h after consumption (Lee et al., 2002; Chow et al., 2003). The EGCG has an absorbed plasma half-life of about 5 h, and most of the unaffected EGCG is excreted into the urine in a period of up to 8 h (Chow et al., 2003). Methylated metabolites seem to have demonstrated harboring longer half-lives than non-methylated compound. (Manach et al., 2005). In 2011, the European Food Safety Authority (EFSA) indicated that a cause-effect connection might not be demonstrated for a link between green tea catechins and the preservation of healthy blood low-density lipoprotein (LDL)-cholesterol rate (EFSA, 2011). Interestingly, five years later, Momose *et al.* unarguably identified that high diurnal EGCG doses ranging from 0.1 to 0.9 g/day administrated to individuals over 4 to 14 weeks promoted some decrease of LDL cholesterol (Momose et al., 2016). Undoubtedly, there are numerous benefits in using EGCG in the diet of a healthy individual and biochemically, and, specifically, cytologically effects have been studied in detail. Cytoprotective effects ascribed to this compound have been identified in the liver, kidney, heart, and lungs (Mezera et al., 2014; Kucera et al., 2015). Nuclear factor erythroid 2 -related factor 2 (NRF2) is a key regulator of the cellular antioxidant response. The induction of NRF2-dependent gene expression seems to be responsible for the protection exercised by this compound in addition to its direct antioxidant effect (Wang et al., 2015; Zhang et al., 2017). There are proofs of activation of glutathione (GSH) S-transferase other than superoxide dismutase and catalase (Augustyniak et al., 2005; Kim et al., 2015). EGCG is also responsible for inhibiting the expression of proinflammatory mediators such as cyclooxygenase 2 (COX-2), inducible nitric oxide synthase (iNOS), tumor necrosis factor α (TNFα) (Oz and Chen, 2008). It also acts in reducing the nuclear factor κB (NF κB) activity (Yang et al., 2001). Byun et al. found that some of the anti-inflammatory actions of EGCG can be attributed to the 67 kDa laminin receptor signaling (Hong Byun et al., 2010). In the setting of its anti-fibrotic effect, EGCG acts by inhibiting the expression of platelet-derived growth factor (PDGF) receptor-β, which is cell a surface tyrosine kinase receptor for members of the PDGF aiming to regulate cell proliferation, cellular differentiation, and cell growth (Zhen et al., 2007). The receptor for insulin-like growth factor 1 (IGF1R) is a transmembrane tyrosine kinase receptor that is activated by a hormone called IGF-1 and by a related hormone (IGF-2) (Sergi et al., 2019), as well as matrix metalloproteinase 2, which is involved in the breakdown of the extracellular matrix. These molecules are also regulated by EGCG in an inhibitory way (Zhen et al., 2007). In the liver, carbon tetrachloride (CCl_4_) can induce fibrosis and EGCG can prevent the development of hepatic fibrosis in rodents (both rats and mice) (Chen et al., 2004; Tipoe et al., 2010). The trigger of *de novo* synthesis of GSH in the hepatic stellate cells of rodent and the interruption of the transforming growth factor β (TGF β) signaling seem to be at the basis of its strong antifibrotic effect on the liver (Yumei et al., 2006). Moreover, EGCG ameliorates the liver tissue affected by non-alcoholic fat liver disease (NAFLD) and non-alcoholic steatohepatitis (NASH) using, for example, the ob/ob mice model, which is a mutant mouse that eats excessively due to mutations in the *LEP* (*Leptin*) gene (Fiorini et al., 2005; Chung et al., 2012). Even the autophagy, which is the regulated mechanism of the cell that removes dysfunctional or unnecessary components naturally (Chiu et al., 2017), is induced by EGCG (Zhou et al., 2014). Attenuation of ischemia/reperfusion injury was found in both healthy (Giakoustidis et al., 2010) and steatotic liver (Fiorini et al., 2005). Overall, the decrease of the risk of cardiovascular diseases seems to be due through its interference with PDGF signaling and, probably, through inhibition of vascular smooth muscle mitogenesis (Lee et al., 2013), by activation of endothelial nitric oxide synthase other than *via* a phosphatidylinositol 3-kinase/Akt-dependent pathway (Lorenz et al., 2004; Potenza et al., 2007), decreased hepatic VLDL secretion, and enhanced biliary secretion of cholesterol (Hirsova et al., 2013).

## Epigallocatechin Gallate Toxicity

In addition to the beneficial effects of GTPs, some studies have emphasized some harmful aspects (Murakami, 2014). In a 2018 review, it was suggested that excessive intake of EGCG might induce some hepatotoxicity (Hu et al., 2018). In the same year, the EFSA stated that a daily intake of 0.8 g might raise the risk of hepatocellular damage (EFSA, 2018). However, it seems that numerous individuals may have an idiosyncratic response to EGCG, and the degree of hepatotoxicity remains the most likely variable among individuals of the same age group. It has been suggested that hepatotoxicity is amplified by genetics, environmental factors, diet, as well as other factors, including age. The US Food and Drug Administration (USFDA) issued a series of reports between 2008 and 2017, targeting some administrative and quality assurance-associated violations by companies commercializing this compound (Wikipedia, 2019). An overdose with EGCG and/or green tea extract can lead to subcellular and cellular injuries (Schmidt et al., 2005; Galati et al., 2006). Pro-oxidative properties have been individuated when the compound is taken at high doses. This pro-oxidative induction is considered to be at the basis of the detrimental effects associated with EGCG. The pro-oxidative activities have been attributed to the catechol structures (*ortho*-diphenols). In pharmacology, an active moiety is the component of a molecule, which is considered to be responsible for either physiological or pharmacological action of a substance. The *ortho*-diphenol is, indeed, the functional moiety, which has the characteristic feature to prompt superoxide anion radical (O_2_^-^) from molecular oxygen (^3^O_2_) using an electrophilic *o*-quinone complement. Moreover, it has been found that an ester group in EGCG may be key in spawning biochemical interactions with the double lipid layer (Kajiya et al., 2001a; Kajiya et al., 2001b). These studies have evidenced a high affinity of this molecular compound for the cell membrane. GTPs covalently bind protein thiols. This covalent bond results from their conversion to an *o*-quinone counterpart. Elbling *et al.* showed that some cellular models may have EGCG-related pro-oxidative but not anti-oxidative properties raising crucial considerations on the diversity of cells, tissues, and organs (Elbling et al., 2005). Overall, the injury can be clarified by the prooxidant effect of high doses of EGCG (Nakagawa et al., 2004; Lambert et al., 2010) as well as by a decrease of both reduced GSH (Galati et al., 2006; Pohanka et al., 2011) and mitochondrial membrane potential (Galati et al., 2006). The single or combined effect induces hepatic necrosis accompanied by an infiltration of polymorphonucleate leukocytes (Goodin et al., 2006).

With regard to the mitochondria, these effects can be intensely argued in the literature. If some authors reported a protective effect of EGCG on mitochondria with a decrease of ROS formation (Jimenez-Lopez and Cederbaum, 2004; Valenti et al., 2013a), others found an inhibitory effect of EGCG on the activity of oxidative phosphorylation enzymes and respiration of mitochondria under ballooning state (Zheng and Ramirez, 2000; Weng et al., 2014). Recently, Kucera et al. (2015) tested the effect of EGCG on primary rat hepatocyte culture and liver mitochondria in permeabilized hepatocytes and found that EGCG promoted decreased ROS production on low doses, but increased ROS formation on high doses. The same authors also supported a decline in mitochondrial membrane potential in cells exposed to EGCG following a comparison with control cells. EGCG seems to determine the damage of the outer mitochondrial membrane and uncoupling of oxidative phosphorylation in permeabilized hepatocytes. EGCG in concentrations lower than 10 μmol/L has been, however, recognized as safe for hepatocytes *in vitro*.

## Infantile and Pediatric Liver

Pre-school and school children may be exposed to numerous compounds that may have toxicity for the liver as the main organ. In consideration of the intricate cytoskeleton assembly and interaction with the extracellular environment the fetal liver development is crucial for a proper function of bile production and excretion and *noxae* to the developing liver may have dramatic consequences (Sergi et al., 2000a; Sergi et al., 2000b; Sergi et al., 2000c; Sergi et al., 2008). From birth through adolescence the developmental changes that occur in the liver's metabolic activity are remarkable and may contribute to the different sensitivity to toxins seen in infants, children, and adolescents (Pineiro-Carrero and Pineiro, 2004). The neonate has one fifth fewer hepatocytes than adult, and this amount is filled from birth through adult when the liver reaches its mature size (Karpen and Suchy, 2001; Wanless, 2002). In the neonate, the capacity to synthesize and excrete bile is immature. This situation gives the neonate a high susceptibility to significant cholestasis from toxic injury. Age-related sensitivity to molecular compounds or drugs is partly attributable to differences in metabolic activity between pediatric and adult liver. If toddlers and children are more resistant to acetaminophen hepatotoxicity when compared with adults, they are more susceptible to toxicity induced from valproic acid, a common anticonvulsant drug (Pineiro-Carrero and Pineiro, 2004). Acetaminophen toxicity is also different from children and adults. There are essential biochemical and subcellular differences between infants/children and adults. In the pediatric liver, sulfation predominates over glucuronidation, which leads to a decreased amount of toxic intermediates. Also, there is a higher capacity to synthesize GSH, thereby inactivating toxic metabolites of acetaminophen more effectively. The enzymatic efficiency is also different between pediatric and adult liver. The postnatal liver shows a decrease of thymidine kinase and ornithine decarboxylase, which is high during intrauterine life.

Conversely, other enzymes, such as fructose-1,6-diphosphatase and aspartate aminotransferase as well as uridine 5-diphosphate glucuronyl transferase, are mildly expressed in the fetus and increase postnatally only. The gray infant syndrome is characterized by the administration of chloramphenicol in an environment with decreased capacity of glucuronide conjugation as seen in the neonatal age (de Wildt et al., 1999). Numerous xenobiotics can be metabolized in the fetal liver, but the neonate exhibits a prolonged half-life for most drugs. It is the first year of life that shows significant and rapid maturation of the liver, and the most rapid elimination of drugs has been determined in school-age children and adolescents; thus, plasma clearance slows with age (Klinger, 2005). The cytochrome P450 system is a cluster of enzymes that control the rate of numerous molecular compounds. Most of these enzymes are located in the liver (hepatic microsomal enzymes) and gut. The main effect of the cytochrome P450 enzymes is to catalyze oxidation, which generally makes the substrate more hydrosoluble and able to be excreted by the kidneys. An alteration of the metabolism of drugs that are inactivated by the cytochrome P450 enzymes can be induced by either induction of these enzymes or the application of compounds that inhibit these enzymes with the final aim to increase the levels of the drug. The total cytochrome P450 (microsomal) content of the liver increases from 0.3 nmol/mg microsomal protein in the fetus/newborn to 0.5 in the full adult. Also, studies of transplacental transfer of aflatoxin B suggest that the developing human liver may be a very sensitive target for aflatoxin B damage (Hakkola et al., 1998; Hukkanen et al., 2002). Although very few data on the function of this enzyme with increasing age are available, it remains valid the concept that adult competence of the microsomal epoxide hydrolase activity is profoundly different between pediatric and adult liver, making infants and children without a doubt at increased risk of drug adverse outcome (Kearns, 1995; Leeder et al., 2010). Overall, it is hypothezable that EGCG may have a remarkable influence on the developing liver. The anti- and pro-oxidative properties of GTPs, as well as many other polyphenols, remain an intriguing field to investigate further. Substantially, both properties have been discovered to be linked to their daily doses, the variable co-presence of oxidants, the cued responsiveness of adaptation systems, and other factors, which are currently under intense investigation.

## “Green Tea”-Associated Liver Disease (GTALD)

**Figure 3:**
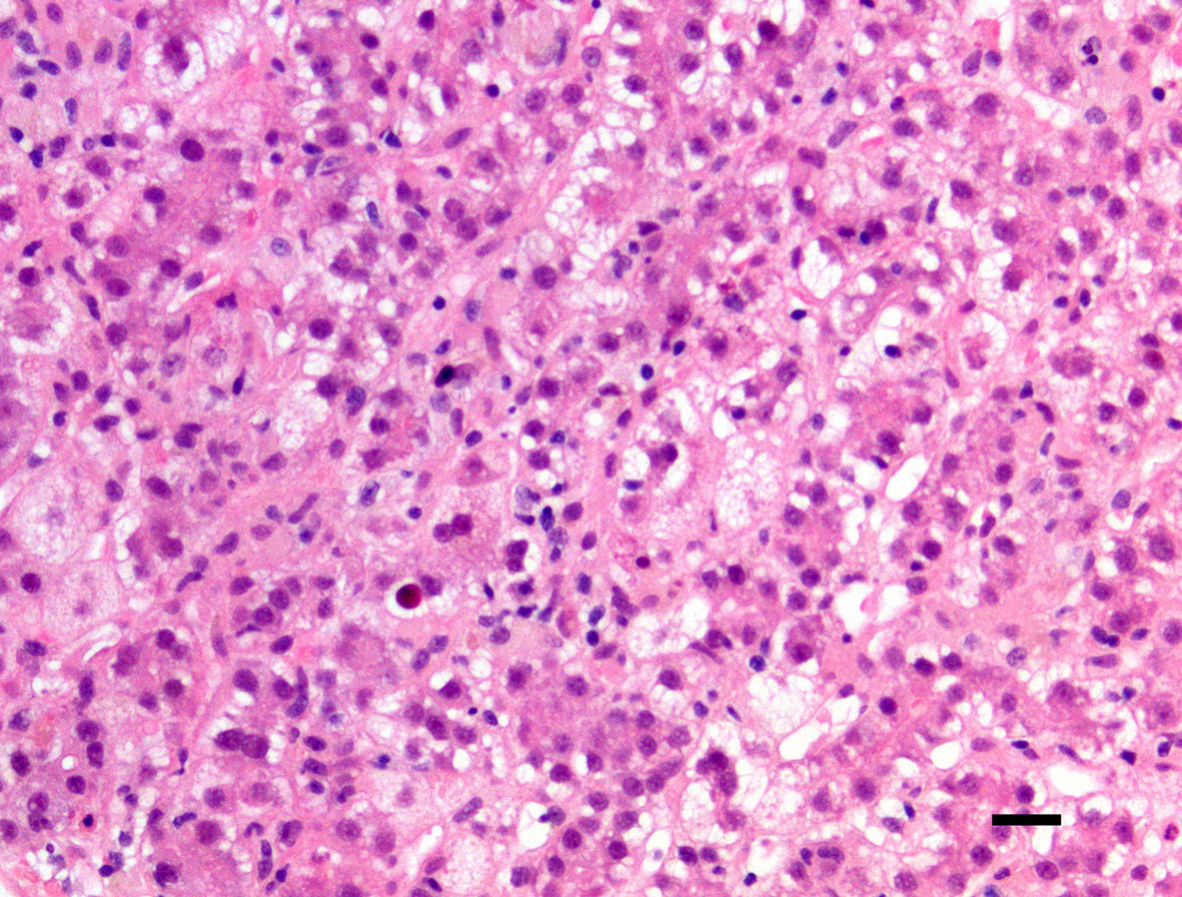
Central lobulus of the liver with hepatocyte ballooning, steatosis, and cell dropout (hematoxylin-eosin staining, ×200 original magnification, bar: 20 micrometers). The patient was a 15-year-old female child with hepatotoxicity due to administered green tea extracts.

**Figure 4:**
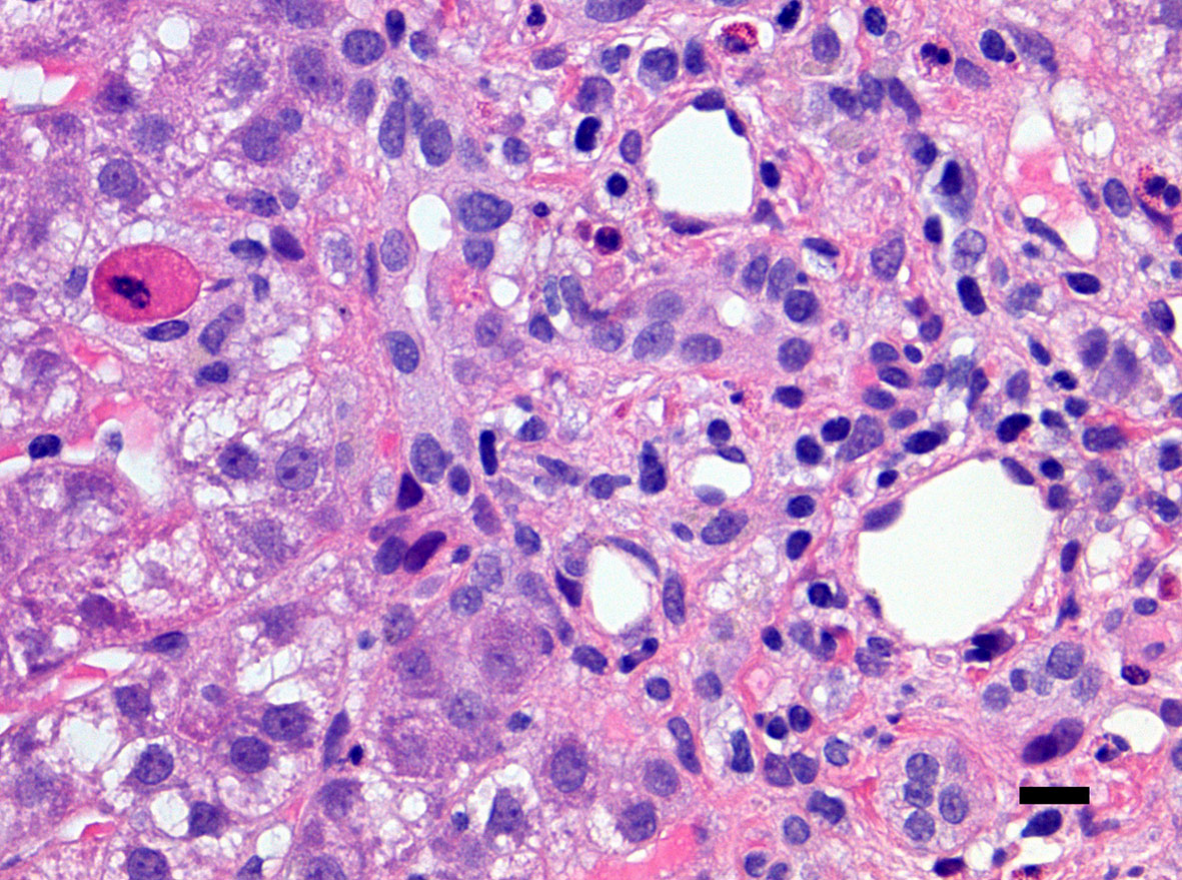
Close-up of a portal tract of the liver of the same patient presented in **Figure 3** including neutrophils and eosinophils with an apoptotic hepatocyte close to the portal tract (left corner of the microphotograph). There is no ductular proliferation, some emperipolesis of inflammatory cells into the bile duct epithelium is noted (right side of the microphotograph) (hematoxylin-eosin staining, ×400 original magnification, bar: 25 micrometers).

Overall, ingestion of high doses of GTPs and other related supplements has been associated with an increased risk of hepatocellular damage because of their pro-oxidative properties. The accurate pathways underlying this toxicity have not been fully elucidated. On the other side, it has been suggested that liver toxicity may exclusively occur with commercial supplements and not by oral consumption of green tea (Mazzanti et al., 2009; Mazzanti et al., 2015). As indicated above, the green tea extract sold in the United States and Western countries is often not the “real” extract from green tea. There is, indeed, an addition of EGCG into the extract of green tea. Thus, the EGCG contents in such GTE products are remarkably higher than the original extract of green tea, and a word of caution should be considered at this time. The impressive increase of traded nutraceuticals of the last two decades in Western countries may raise the awareness that some individuals may be particularly susceptible to EGCG and related compounds. Following the biochemical considerations detailed above, it seems that “green tea”-associated liver disease (GTALD) is mostly due to hepatocellular damage. In 15 out of the 19 cases reviewed by Mazzanti et al. (2015), a liver biopsy was performed. Liver histopathology showed mostly necrosis ranging from submassive, to massive or multifocal and accompanied by widespread inflammatory cellular infiltrates. The hepatocellular necrosis may evidence multilobular collapse of all three Rappaport hepatocytic zones (zone 1, periportal, zone 2 midlobular, and zone 3 pericentral). Livers with massive hepatic necrosis disclose a change in color and shape, exhibiting a shrinkage and wrinkling of the capsule. The cut surface of the liver is homogeneous with some mottling of the hepatic parenchyma. This aspect seems to be due to the intermingling of red (hyperemic) and yellow (steatotic) areas. In some organs, the necrotizing phenomenon may spare few regions of the liver parenchyma. This advance of disease may leave irregular islands of tissue or nodular clusters amongst collapsed parenchyma. Occasionally, cholestasis and mild fibrosis can be observed. Macroregenerative nodules need to be histologically investigated, although it does not seem that a hepatocellular carcinoma has been ever diagnosed in GTALD. According to Danan and Benichou (Danan and Benichou, 1993), the type of liver damage should be classified as hepatocellular in the majority of the cases (84.2%) and as cholestatic in two cases (10.5%). In **Figures 3** and **4**, is shown the liver histopathology with GTP-associated damage identified in a child whose nutrition was complemented with green tea extracts. It is important to reiterate at this time that pathology evidencing FHF is commonly non-specific and often points to viruses, autoimmunity, or congenital disorders of metabolism. A meticulous medical and drug history remains key representing an important task provided by pediatric gastroenterologists and hepatologists. The differential diagnosis between virus-triggered and toxic compounds-associated FHF relies on the first instance on a good medical history, indagating not only the pharmacological history but also the habit and the attitudes of the child or adolescent and both parents.

## Final Remarks and Conclusion

In internal medicine, cardiovascular disease, hypertension, hyperlipidemia, and diabetes mellitus are jointly known as “metabolic syndrome.” Also, there is evidence indicating that overweight and its related metabolic abnormalities are linked to an individual predisposition to develop some types of human epithelial malignancies, which score high in cancer statistics, including colorectal cancer and hepatocellular carcinoma (Shimizu et al., 2013; Shimizu et al., 2012). There is some pathophysiology linking overweight and tumorigenesis. It includes the emergence of insulin resistance, other than variations in the insulin-like growth factor-1 (IGF-1)/IGF-1 receptor (IGF-1R) axis, occurrence of adipocytokine imbalance, the state of chronic tissue inflammation, and the induction of cellular oxidative stress (Shimizu et al., 2012; Yang et al., 2018; Sergi et al., 2019). Green tea catechins have probably attracted considerable attention in the last two decades. The beneficial effects of improving metabolic abnormalities and preventing cancer growth are mostly based on tremendous interest worldwide. Although the use of nutraceuticals has been used in obese or overweighted adults, it should not be considered in childhood because of the potential adverse effect on immature or fast-developing liver cells. Hepatotoxicity and histologically confirmed hepatitis due to GTPs in children are probably very rare because of the occasional use, but these events may become more frequent in the future, because of the growing interest and/or change of the attitude toward botanicals. We must emphasize that liver failure remains a challenge for the pediatric gastroenterologist and nutritionist who need to stay knowledgeable in several fields of basic and clinical hepatology and hepatic pharmacology.

## Author Contributions

CS conceptualized and designed the study, performed numerous clinical and surgical pathology procedures, collected data, carried out the histopathological analysis of innumerable liver samples with fulminant hepatic failure of various etiology, and revised the manuscript.

## Funding

This research has been funded by the generosity of the Stollery Children’s Hospital Foundation and supporters of the Lois Hole Hospital for Women through the Women and Children’s Health Research Institute, Hubei Province Natural Science Funding for Hubei University of Technology (100-Talent Grant for Recruitment Program of Foreign Experts Total Funding: Digital PCR and NGS-based diagnosis for infection and oncology, 2017-2022), Österreichische Krebshilfe Tyrol (Krebsgesellschaft Tirol, Austrian Tyrolean Cancer Research Institute), Austrian Research Fund (Fonds zur Förderung der wissenschaftlichen Forschung, FWF), Canadian Foundation for Women’s Health, Cancer Research Society (von Willebrand factor gene expression in cancer cells), Canadian Institutes of Health Research (Omega-3 Fatty Acids for Treatment of Intestinal Failure Associated Liver Disease: A Translational Research Study), and the Saudi Cultural Bureau, Ottawa, Canada. The funders had no role in study design, data collection and analysis, decision to publish, or preparation of the manuscript.

## Conflict of Interest

The author declares that the research was conducted in the absence of any commercial or financial relationships that could be construed as a potential conflict of interest.
